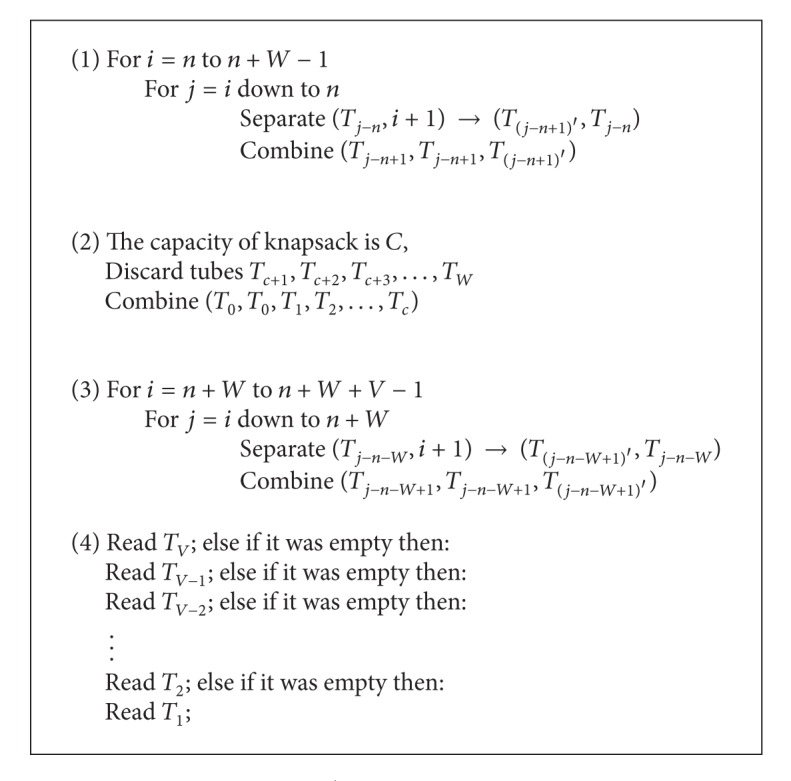# Solving the 0/1 Knapsack Problem by a Biomolecular DNA Computer

**DOI:** 10.1155/2013/341419

**Published:** 2013-02-18

**Authors:** Hassan Taghipour, Mahdi Rezaei, Heydar Ali Esmaili

**Affiliations:** ^1^Department of Pathology, Tabriz University of Medical Sciences, Tabriz, Iran; ^2^Department of Theoretical Physics and Astrophysics, University of Tabriz, Tabriz 51664, Iran

## Abstract

Solving some mathematical problems such as NP-complete problems by conventional silicon-based computers is problematic and takes so long time. DNA computing is an alternative method of computing which uses DNA molecules for computing purposes. DNA computers have massive degrees of parallel processing capability. The massive parallel processing characteristic of DNA computers is of particular interest in solving NP-complete and hard combinatorial problems. NP-complete problems such as knapsack problem and other hard combinatorial problems can be easily solved by DNA computers in a very short period of time comparing to conventional silicon-based computers. Sticker-based DNA computing is one of the methods of DNA computing. In this paper, the sticker based DNA computing was used for solving the 0/1 knapsack problem. At first, a biomolecular solution space was constructed by using appropriate DNA memory complexes. Then, by the application of a sticker-based parallel algorithm using biological operations, knapsack problem was resolved in polynomial time.

## 1. Introduction

DNA encodes the genetic information of cellular organisms. The unique and specific structure of DNA makes it one of the favorite candidates for computing purposes. In comparison with conventional silicon-based computers, DNA computers have massive degrees of miniaturization and parallelism. By recent technology, about 10^18^ DNA molecules can be produced and placed in a medium-sized laboratory test tube. Each of these DNA molecules could act as a small processor. Biological operations such as hybridization, separation, setting, and clearing can be performed simultaneously on all of these DNA strands. Thus, in an in vitro assay, we could handle about 10^18^ DNA molecules or we can say that 10^18^ data processors can be executed in parallel.

In 1994, Adleman introduced the DNA computing as a new method of parallel computing [1]. Adleman succeeded in solving seven-point Hamiltonian path problem solely by manipulating DNA molecules and suggested that DNA could be used to solve complex mathematical problems.

In 1999, a new model of DNA computing (sticker model) was introduced by Roweis et al. [2]. This model has a kind of random access memory that requires no strand extension, uses no enzymes, and its materials are reusable. Sticker-based DNA computing has potential capability for being a universal method in DNA computing. Roweis et al. [2] also proposed specific machine architecture for implementing the sticker model as a microprocessor-controlled parallel robotic workstation. Thus, the operations used in sticker model can be performed on fully automated devices, which is helpful in reducing the error rates of operations.

In this paper, we applied sticker model for solving the knapsack problem which is one of the NP-complete problems.

The paper is organized as follows.  Section 2 introduces the DNA structure and various DNA computing models and discusses about the sticker based DNA computing and biological operations which are used in sticker model.  Section 3 introduces a DNA-based algorithm for solving the knapsack problem in sticker model.

## 2. Basics of DNA Computing

### 2.1. Structure of DNA and DNA Computing Models

**Figure 1:**
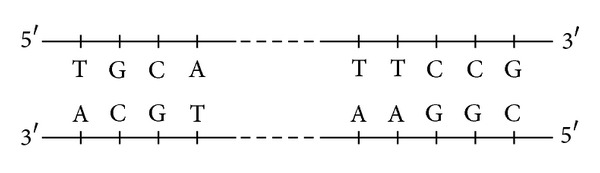
A DNA molecule.

DNA is a polymeric and a double-stranded molecule which is composed of monomers called nucleotides. Nucleotides are building blocks of DNA, and each of them contains three components: sugar, phosphate group, and nitrogenous base. There are four different nitrogenous bases which contribute in DNA structure: Thymine (T) and Cytosine (C) which are called pyrimidines and Adenine (A) and Guanine (G) which are called purines. Because nitrogenous bases are variable components of nucleotides, different neucleotides are distinguished by nitrogenous bases which contribute in their structure. For this reason, the name of the bases are used to refer to the neucleotides, and the neucleotides are simply represented as A, G, C, and T. The nucleotides are linked together by phosphodiester bonds and form a single-stranded DNA (ssDNA). A ssDNA molecule can be likened to a string consisting of a combination of four different symbols, A, G, C, and T. Mathematically, this means that we have a four-letter alphabet ∑ = {A, G, C, T} to encode information. Two ssDNA molecules join together to form a double-stranded DNA (DsDNA) based on complementary rule: “A” always pairs with “T,” and likewise “C” pairs with “G.” In Figure 1, a schematic picture of DNA is shown.

DNA computing was initially developed by Adleman in 1994. Adleman resolved an instance of Hamiltonian path problem just by handling the DNA molecules [1]. In 1995, Lipton presented a method for solving the satisfiability (SAT) problem [3]. Adleman-Lipton model can be used to solve different NP-complete problems. In Adleman-Lipton model, DNA splints are used for the construction of solution space. Adleman [4, 5] also presented a molecular algorithm for solving the 3-coloring problem. Chang and Guo [6–8] showed that the DNA operations in Adelman-Lipton model could be used for developing DNA algorithms to resolve the dominating set problem, the vertex cover problem, the maximal clique problem, and the independent set problem.

In 1999, Roweis et al. [2] introduced the Sticker based DNA computing model and applied it in solving the minimal set cover problem, and this model also was applied for breaking the Data Encryption Standard (DES) [9]. In our previous work, we also applied sticker based model for solving the independent set problem [10].

Other than Adleman-Lipton and Sticker based models, other various models are also proposed in DNA computing by researchers. Quyang et al. [11] solved the maximal clique problem using DNA molecules and restriction endonuclease enzymes. Amos et al. [12, 13] described a DNA computation model using restriction endonuclease enzymes instead of successive cycles of separation by DNA hybridization, which can reduce the error rate of computation. Hagiya et al. [14] proposed a new method of DNA computing that involves a self-acting DNA molecule containing both the input, program, and working memory. In this method, a single-stranded DNA molecule consists of an input segment on the 5′ end, followed by a formula (program) segment, followed by a spacer, and finally with a “head” on the 3′ end that moves and performs the computation. Another method for DNA computation is “computation by self-assembly.” Winfree et al. [15–17] introduced a linear and 2-dimensional self-assembly model.

The surface-based model was introduced by Liu et al. [18]. This model uses DNA molecules attached to a solid surface, instead of DNA molecules floating in a solution. The surface-based model was used by Taghipour et al. for solving the dominating set problem [19]. The computing by blocking was introduced by Rozenberg and Spaink [20]. This model uses a novel approach to filter the DNA molecules. Instead of separating the DNA strands to distinct tubes, or destroying and removing the DNA molecules that do not contribute to finding a solution, it blocks (inactivates) them in a way that the blocked strands can be considered as nonexistent during the subsequent steps of computation.

### 2.2. Sticker-Based DNA Computation

The sticker model was introduced by Roweis et al. [2]. In this model, there is a memory strand with *N* bases in length subdivided into *K* nonoverlapping regions each *M* bases long (*N* ≥ *MK*). *M* can be, for example, 20. The substrands (bit regions) are significantly different from each other. One sticker is designed for each subregion; each sticker has *M* bases long and is complementary to one and only one of the *K* memory regions. If a sticker is annealed to its corresponding region on memory strand, then the particular region is said to be *on*. If no sticker is annealed to a region, then the corresponding bit is *off*. Each memory strand along with its annealed stickers is called memory complex. In sticker model, a tube is a collection of memory complexes, composed of large number of identical memory strands each of which has stickers annealed only at the required bit positions. This method of representation of information differs from other methods in which the presence or absence of a particular subsequence in a strand corresponded to a particular bit being *on* or *off*. In sticker model, each possible bit string is represented by a unique association of memory strands and stickers. This model has a kind of random access memory that requires no strand extension and uses no enzymes [2]. Indeed, in the sticker model, memory strands are used as registers, and stickers are used to write and erase information in the registers.

**Figure 2:**
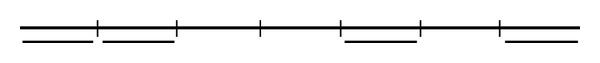
A memory complex representing 1100101.

Another conception in sticker model is (*K*, *L*) library. Each (*K*, *L*) library contains memory complexes with *K* bit regions, the first *L* bit regions are either on or off, in all possible ways, whereas the remaining *K*-*L* bit regions are off. The last *K*-*L* bit regions can be used for intermediate data storage. In every (*K*, *L*) library, there are at least 2^*L*^ memory complexes. In Figure 2, a memory complex with 7 bit regions representing the binary number 1100101 is shown.

### 2.3. Biological Operations in Sticker Model

There are four principal operations in sticker model: combination, separation, setting, and clearing [2]. We also defined a new operation called “divide” which is used in the construction of solution space [10]. Here, we briefly discuss about these operations.

1. *Combine *(*T*_0_, *T*_1_, and *T*_2_). The memory complexes from the tubes *T*_1_ and *T*_2_ are combined to form a new tube, *T*_0_, simply the contents of *T*_1_ and *T*_2_ are poured into the tube *T*_0_. (*T*_0_ = *T*_1_ ∪ *T*_2_).
2. *Separate *(*T*_0_, *i*)→(*T* ^+^, *T* ^−^). This operation creates two new tubes *T* ^+^ and *T* ^−^; *T* ^+^ contains the memory complexes having the *i*th bit on (*T* ^+^ = + (*T*_0_, *i*)), and *T* ^−^ contains the memory complexes having the *i*th bit off (*T* ^−^ = − (*T*_0_, *i*)).
3. *Set *(*T*_0_, *i*). The *i*th bit region on every memory complex in tube *T*_0_ is set to 1 or turned on.
4. *Clear *(*T*_0_, *i*). The *i*th bit region on every memory complex in tube *T*_0_ is set to 0 or turned off.
5. *Divide* (*T*_0_, *T*_1_, and *T*_2_). By this operation, the contents of tube *T*_0_ is divided into two equal portions and poured into the tubes *T*_1_ and *T*_2_.

## 3. Solving the 0/1 Knapsack Problem in Sticker-Based DNA Computers

### 3.1. Definition of the Knapsack Problem

Knapsack problem is one of the classical optimization problems which have two variants: the 0/1 and fractional knapsack problems.

The 0/1 knapsack problem is posed as follows.

There are *n* items *I*_1_, *I*_2_, *I*_3_,…, *I*_*n*_; each item *I*_*j*_ has a weight *W*_*j*_ and a value *V*_*j*_, where *W*_*j*_ and *V*_*j*_ are integers. We have a knapsack which its capacity (weight) is *C*, where *C* is also an integer. We want to take the most valuable set of items that fit in our knapsack. Which items should we take? This is called the 0/1 or binary knapsack problem because each item must either be taken or left behind; we cannot take a fractional amount of an item.

In the fractional knapsack problem, the setup is the same, but we can take fractions of items, rather than having to make a binary (0-1) choice for each item. The fractional knapsack problem is solvable by a greedy strategy, where as the 0/1 knapsack problem is not. The 0/1 knapsack problem has been proved to be an NP-complete problem [21].

### 3.2. Construction of Sticker Based DNA Solution Space for Knapsack Problem

#### 3.2.1. Designing Appropriate DNA Memory Complexes

As discussed before, there are *n* items *I*_1_, *I*_2_, *I*_3_,…, *I*_*n*_; each item *I*_*j*_ has a weight *W*_*j*_ and a value *V*_*j*_, where *W*_*j*_ and *V*_*j*_ are integers. Let us consider that the total weight of items is *W* and total value of items is *V*.

(1) $$\begin{array}{c} W=W_{1}+W_{2}+W_{3}+\cdots +W_{n}=\sum_{j=1}^{n}W_{j},\\ V=V_{1}+V_{2}+V_{3}+\cdots +V_{n}=\sum_{j=1}^{n}V_{j} \end{array}$$

*n* = total number of items.

**Figure 3:**
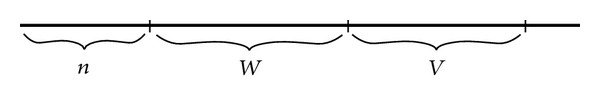
Memory strand with at least *n* + *W* + *V* bit regions.

We start with 2^*n*^ or more identical memory strands, which each of them has at least *n* + *W* + *V* bit regions. (Figure 3) The first *n* bit regions (bit regions 1 to *n*) are used to represent *n* items, the middle *W* bit regions (bit regions *n* + 1 to *n* + *W*) represent the total weight of items *W*, and the next *V* bit regions (bit regions *n* + *W* + 1 to *n* + *W* + *V*) represent the total value of items *V*. Each bit region, for example can have 20 neucleotides, furthermore, every memory strand at least contains 20 (*n* + *W* + *V*) neucleotides.

#### 3.2.2. Production of DNA Memory Complexes Which Represent All Possible Subsets of Items

It is clear that a set of *n* items has 2^*n*^ subsets and each of these subsets has its own weight and value. For construction of solution space, it is essential to represent all subsets of items by appropriate DNA memory complexes. Furthermore, by using at least 2^*n*^ or more memory strands and making the first *n* bit regions *on* or *off *in all possible ways, we represent all 2^*n*^ subsets of items by DNA memory complexes. On the other hand, simply we design a (*n* + *W* + *V*,  *n*) library. For this purpose, (Procedure 1) is proposed.

Procedure 1 has *n* divide, *n* set and *n* combine operations. At the end of procedure, tube *T*_0_ contains all of the memory complexes which each of them represent one of the subsets of items.

#### 3.2.3. Representing the Weight and Value of Each Subset on DNA Memory Complexes

In this step, based on the items which are present in subsets, and by annealing corresponding stickers in *W* and *V* regions of memory strands, the total weight and value of subsets are represent on memory complexes. Note, each item *I*_*j*_ has a weight *W*_*j*_ and a value *V*_*j*_, thus, for each item *I*_*j*_, *W*_*j*_ numbers of stickers are annealed to *W* region and *V*_*j*_ numbers of stickers are annealed to *V* region on memory strands. Furthermore, the numbers of annealed stickers in *W* and *V* regions represent the weight and value of corresponding subset, respectively. Procedure 2 is proposed for representing the weight and value of each subset.

Now, our solution space is completely produced and contains at least 2^*n*^ memory complexes, which each of them represent one of the subsets of items, and the numbers of annealed stickers in *W* and *V* regions represent the weight and value of corresponding subset, respectively.

### 3.3. DNA Algorithm for Solving the 0/1 Knapsack Problem

Algorithm 1 is proposed for solving the 0/1 knapsack problem.

According to the steps in the algorithm, the knapsack problem can be resolved by sticker based DNA computation in polynomial time.

By the execution of step 1, the memory complexes without any annealed stickers in *W* region (represent the subset *∅*) are placed in tube *T*_0_, the memory complexes with only one annealed sticker (represent the subsets of items which their weight are 1) are placed in tube *T*_1_, the memory complexes with 2 annealed stickers (represent the subsets of items which their weight are 2) are placed in tubes *T*_2_, the memory complexes with 3 annealed stickers (represent the subsets of items which their weight are 3) are placed in tube *T*_3_, and finally, the tube *T*_*W*_ contains the memory complexes witch all bit regions located in *W* region are turned to “on” (represent the subset which contains all items). On the other hands, step 1 is a sorting procedure and sorts memory complexes according to the number of annealed stickers in *W* region. In this step, *W* + 1 tubes are produced (*T*_0_, *T*_1_, *T*_2_,…, *T*_*W*_), and number of every tube indicate the number of annealed stickers in *W* region. Step 1 contains *W*(*W* + 1)/2 separate and  *W*(*W* + 1)/2 combine operations, or totally it contains *W*(*W* + 1) operations.

In step 2 of algorithm, the contents of tubes *T*_*c*+1_, *T*_*c*+2_, *T*_*c*+3_,…, *T*_*W*_ are discarded, because memory complexes which are present in these tubes, represent subset of items that their weight are exceeded the capacity of knapsack. Then, the contents of tubes *T*_0_, *T*_1_, *T*_2_,…, *T*_*c*_ are mixed together and transferred to tube *T*_0_. Now, tube *T*_0_ contains memory complexes which represent the subsets of items that their weight are not exceeded the capacity of knapsack. Furthermore, at the end of step 2, the memory complexes which represent the subsets of items that their weight are exceeded the capacity of knapsack, removed from solution space and only remain memory complexes representing subsets that fit in our knapsack. It is clear that the step 2 contains only 2 operations.

By the execution of step 3, sorting of memory complexes are performed according to the number of annealed stickers in *V* region. During this step, *V* + 1 tubes are produced (*T*_0_, *T*_1_, *T*_2_,…, *T*_*V*_). The memory complexes without any annealed stickers in *V* region (represent the subset *∅*) are placed in tube *T*_0_, the memory complexes with only one annealed sticker (represent the subsets of items which their value are 1) are placed in tube *T*_1_, the memory complexes with 2 annealed stickers (represent the subsets of items which their value are 2) are placed in tubes *T*_2_, the memory complexes with 3 annealed stickers (represent the subsets of items which their value are 3) are placed in tube *T*_3_, and finally, the tube *T*_*V*_ contains the memory complexes with all bit regions located in *V* region are turned to “on” (represent the subset which contains all items). Step 3 contains *V*(*V* + 1)/2 separate and *V*(*V* + 1)/2 combine operations, or totally it contains *V*(*V* + 1) operations.

In step 4, all of tubes (from *T*_*V*_ to *T*_1_) are evaluated for presence of memory complexes, and the first tube which is not empty and contains memory complexes represent the most valuable set. Step 4, maximally contains *V* Read operations.

Finally, it is clear that the total number of operations in our algorithm is: *W* ^2^ + *V* ^2^ + *W* + 2∗*V* + 2.

## 4. Conclusion

In this paper, the sticker based DNA computing was used for solving the 0/1 knapsack problem. This method could be used for solving other NP-complete problems. There are four principal operations in sticker model: Combination, Separation, Setting and Clearing. We also defined a new operation called “divide” and applied it in construction of solution space.

As mentioned earlier, one of the important properties of DNA computing is its real massive parallelism, which makes it a favorite and powerful tool for solving NP-complete and hard combinatorial problems. In sticker model, as in other DNA based computation methods, the property of DNA molecules to making duplexes is used as main biological operation. The main difference between the sticker model and Adleman-Lipton model is that in the sticker model there is a kind of Random access memory and the computations do not depend on DNA molecules extension as seen in Adleman-Lipton model.

## Figures and Tables

**Procedure 1:**
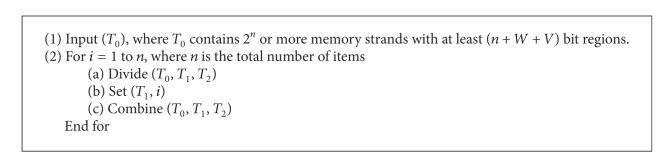


**Procedure 2:**
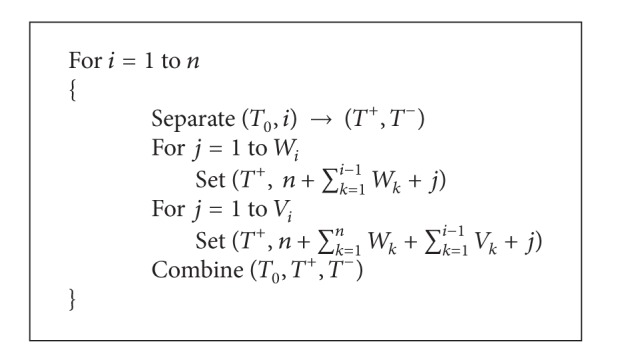


**Algorithm 1:**